# Genetic diversity of the *RHD* gene in blood donors from the Brazilian Central-West

**DOI:** 10.1016/j.htct.2026.106506

**Published:** 2026-07-28

**Authors:** Ana Luisa Alves Mafra, Wagner Ribeiro de Mesquita, Barbara Maciel Sidou Pimentel, Fabiola Gonçalves Ulhoa Andre, Flávia Leite Souza Santos, Dimas Tadeu Covas, Lilian Castilho, Simone Kashima, Evandra Strazza Rodrigues

**Affiliations:** a Laboratório de Imuno-hematologia de Doadores da Fundação Hemocentro de Brasília (FHB), Distrito Federal, Brazil; b Fundação Hemocentro de Ribeirão Preto, Faculdade de Medicina de Ribeirão Preto, Universidade de São Paulo (USP), Ribeirão Preto, Brazil; c Laboratório de Imuno-hematologia Molecular do Hemocentro de Campinas – Universidade de Campinas (UNICAMP), Campinas, Brazil

**Keywords:** Rh system, D antigen, RHD variants, Brazilian blood donors

## Abstract

**Introduction:**

The Rh blood group system is recognized for its complexity and its clinical importance in transfusion medicine. Antibodies against Rh antigens are associated with hemolytic transfusion reactions, autoimmune hemolytic anemia, and hemolytic disease of the fetus and newborn. The present study aims to characterize D antigens and assess their allele frequencies at the molecular level.

**Methods:**

Molecular characterization of *RHD* variants was performed on blood donors from the Brazilian Central-West who presented atypical D typing results. Serological profiles of all identified *RHD* variant alleles were also analyzed using different anti-D clones.

**Results:**

Among the D-positive samples, 1.25% exhibited weak or discrepant agglutination during serological D typing. Most samples (67/103; 65%) were classified within the RHD*weak partial 4 cluster, followed by RHD*weak D type 3 (17/103; 16%). Lower frequencies were observed for RHD*weak D type 1 (2/103; 1.9%) and RHD*weak D type 2 (6/103; 5.8%). Furthermore, the rare RHD*weak D type 38 and RHD*weak D type 145 variants were identified. Analysis with different anti-D reagents showed that 52% of the samples had agglutination scores below 2+. Notably, 27% were classified as inconclusive, and 21% exhibited RhD serological discrepancies.

**Conclusion:**

Atypical reactions in RhD serological testing indicate the presence of variant D antigens. The RHD*weak partial 4 allele was the most prevalent *RHD* variant in the Brazilian Central-West population. However, the identification of rare RHD*weak D variants, such as types 38 and 145, highlight the genetic diversity reflective of the region’s multiracial composition. Understanding the distribution of *RHD* variants can improve transfusion support and inform future *RHD* genotyping strategies.

## Introduction

The Rh blood group system (ISBT 004) is recognized for its complexity and its clinical importance in transfusion medicine. Antibodies against Rh antigens are associated with hemolytic transfusion reactions (HTRs), autoimmune hemolytic anemia (AIHA), and hemolytic disease of the fetus and newborn (HDFN) [1,2].

The clinical relevance of the Rh system was first identified by Levine and Stetson in 1939, during the investigation of a severe HTR in a puerperal woman who had received ABO-compatible blood from her husband following the birth of a stillborn baby. The antibody present in the woman’s serum, despite her lack of prior transfusion, agglutinated the red blood cells (RBCs) of her husband and of approximately 80% of ABO-compatible blood donors [3,4]. At the time, this unknown antigen was presumed to have caused alloimmunization due to fetal antigenic exposure of paternal origin. The hemolytic episodes were attributed to the maternal antibody response against the antigen present on the transfused RBCs [5]. Subsequently, this antigen was named Rh, and its main antigenic components (C, c, E, and e) were identified [6]. Today, the detection of Rh antibodies is an essential component of transfusion services and prenatal care, playing a critical role in preventing HTRs and HDFN.

In Brazil, the determination of the D antigen in blood donors is performed using serological testing with monoclonal anti-D antisera. When the initial D typing yields a negative result, testing for weak D antigens is performed. For this analysis, it is recommended to use at least two different anti-RD reagents, with at least one containing IgG antibodies. If either the D typing or the weak D antigen test is positive, the donor’s blood is labeled ‘RhD positive’ and is released for transfusion. Blood is classified as RhD-negative only when both the initial D typing and the subsequent weak D test yield negative results [7].

Although serological phenotyping remains the standard method for D antigen analysis, molecular *RHD* genotyping is recommended when discrepancies between anti-D reagents are observed or when reduced D antigen expression is observed during D typing. This limitation arises because standard serological techniques are often unable to accurately distinguish between D antigen variants, such as weak D and partial D phenotypes. These variants may exhibit weak or inconsistent agglutination patterns that are not conclusively resolved by standard testing algorithms. Consequently, molecular genotyping has become an essential tool for the accurate classification of D variants and for guiding appropriate clinical management [8,9].

The prevalence and distribution of *RHD* variant alleles vary markedly across populations. Studies demonstrate a predominance of weak D types 1–3 among individuals of European ancestry; importantly, most carriers of these variants are unlikely to develop alloanti-D following exposure to D-positive RBCs. Accordingly, current clinical guidelines recommend that such individuals be managed as D-positive, permitting transfusion with D-positive RBCs and obviating the need for Rh immune globulin (RhIG) prophylaxis [10,11].

In contrast, other populations, including individuals of African descent and cohorts from Nigeria [12], Iran [13], and Brazil [14], exhibit distinct *RHD* allele distributions, characterized by a higher prevalence of partial D variants. These variants may be associated with an increased risk of alloanti-D formation [12]. Therefore, *RHD* genotyping is strongly recommended, particularly in individuals typed as D positive who present with anti-D antibodies.

In ethnically admixed populations such as Brazil, molecular studies have revealed a complex spectrum of *RHD* variants that differs from those typically observed in predominantly Caucasian populations, with appreciable frequencies of both weak D and partial D alleles [15]. Studies conducted in southeastern Brazil have identified *RHD* weak partial type 4 as one of the most prevalent variants among blood donors [14], whereas in southern Brazil, weak D types 1 and 4 have been reported as the most frequent variants [16,17].

Notably, the Brazilian population requires particular attention due to its extensive ethnic admixture combined with a large territorial expanse. This unique demographic context reflects a highly heterogeneous genetic background shaped by centuries of migration and admixture, contributing to significant regional variability in the distribution of *RHD* alleles. Moreover, data on the distribution and frequency of *RHD* variants in Brazil remain limited, with most studies concentrated in the southeastern region. Expanding investigations to other regions is essential, as historical processes, including Portuguese, Italian and African immigration, have differentially influenced the genetic composition of the population, potentially impacting the regional distribution of *RHD* variants. In this context, the present study aims to characterize D antigens and assess their allele frequencies at the molecular level, focusing on blood donors from the Fundação Hemocentro de Brasília, located in the Federal District, Central-West region of Brazil.

## Material and methods

### Ethics considerations

The study was approved by the Research Ethics Committee of the Blood Bank of Brasília, DF and Health Secretariat of the Federal District, Brazil (CEP/SES/DF, protocol N°CAAE: 60130416.0.0000.5553). The Blood Bank of Ribeirão Preto (FUNDHERP) was included as a co-participating institution.

### Subjects and sample collection

In this prospective study, 103 peripheral blood samples from the Blood Bank of the Federal District, Brazil, were subjected to molecular analysis of the *RHD* gene. These samples were selected from a total of 8,240 voluntary blood donors who were initially screened using a serological D assay. The samples were collected between October and December 2016. All eligible donors were included, with exclusions restricted to samples presenting hemolysis, insufficient volume, or incomplete data. This sample size provides sufficient power to estimate the frequency of variant D alleles within a 95% confidence interval.

Genomic DNA was extracted from approximately 5 mL of whole blood collected in ethylenediaminetetraacetic acid (EDTA) tubes (Vacuette, Greiner Bio-One). The samples were obtained from individuals who exhibited inconclusive or weak agglutination reactions (less than 2+) for the D antigen during routine direct D typing. The weak D phenotype was subsequently confirmed by the indirect antiglobulin test (IAT). All samples were stored under refrigeration (4–8°C) until DNA extraction.

### DNA extraction

Genomic DNA was extracted from peripheral blood using the Biopur Mini Spin Plus 250 kit (Mobius Life Science, Pinhais – PR, BR) according to the manufacturer's instructions. The concentration and quality of the DNA were analyzed by optical density in a spectrophotometer (NanoDrop Lite, Thermo Scientific).

### RHD genotyping

All samples were screened for the presence or absence of *RHD* by analyzing two genomic regions, intron 4 and exon 7, as previously described [18]. *RHD* molecular analyses for weak D types 1, 2, 3, and 4 were performed by single specific primer-polymerase chain reaction (PCR-SSP). Specific primers were used for the PCR-SSP amplification reaction as previously described [19]. The reactions were performed in a total volume of 25 µL containing 100 ng of DNA, 1.0 U of Taq DNA polymerase (Invitrogen, California, USA), 50 mM KCl, 20 mM Tris-HCl, pH 8.3, 1.5 mM MgCl_2_, 0.2 mM deoxynucleotide triphosphate (dNTP), and 0.3 pmol of each specific primer. Thermocycling was performed in the GeneAmp PCR system 9700 (Perkin-Elmer/Cetus, Connecticut, USA). Polymerase chain reaction (PCR) cycling conditions consisted of an initial cycle of 2 min at 94°C, followed by 35 cycles of 40 seconds at 94°C, 1 min at 56°C, 2.3 min at 72°C, and a final extension at 72°C for 10 min. The amplified products were analyzed by 1% agarose gel electrophoresis and visualized with GelRed staining (Biotium Corporate Headquarters, California, USA) (Supplementary Fig. 1). RHCE*Cc and RHCE*Ee genotyping was performed as previously described [20]. For each molecular analysis, positive and negative controls were performed by replacing genomic DNA with previously genotyped DNA or DNase/RNase-free H_2_O (Invitrogen, CA, USA), respectively.

### Sanger sequencing and RHD variants analysis

Genomic DNA sequencing was performed as previously described [21] to confirm PCR results and to determine the less prevalent *RHD* alleles. Sanger sequencing was performed using the Big Dye Terminator version 3.1 cycle sequencing kit (Applied Biosystems) on the ABI PRISM 3100-Avant Genetic Analyzer (Applied Biosystems). Comprehensive sequencing of all *RHD* coding exons and flanking intronic regions enabled the detection of splicing mutations, precise classification of *RHD* variants, and identification of rare alleles through comparison with reference sequences (Supplementary Figure 2). Occasional exon-specific amplification failures were attributed to incomplete PCR, potential gene rearrangements or unclassified *RHD* alleles.

### Serological phenotyping

RhD phenotyping was performed using the hemagglutination method in U-bottom microplates containing lyophilized monoclonal anti-D antibodies from two distinct cell lines, TH-28 (IgM) and MS-201/MS-26 (IgM/IgG) (DiaMed GmbH, Cressier FR, Switzerland, Biorad®). The reactions were read automatically using the Lyra equipment (Biorad®).

All samples that exhibited negative, inconclusive, or weak agglutination (less than 2+) for the D antigen were subjected to weak D confirmation testing. This confirmation was performed using the IAT at 37°C, utilizing a Coombs gel card containing rabbit anti-IgG antibodies (Bio-Rad, Cressier, Switzerland). Rh phenotyping for C, c, E, e antigens was performed using the gel card method with the following monoclonal antibodies: anti-C (clone MS-24), anti-c (clone MS-33), anti-E (clone MS-260), anti-e (clones MS-16, MS-21, MS-63 (Biorad® Cressier FR, Switzerland). All serological tests, including weak D confirmation and extended Rh phenotyping, were conducted automatically using the Techno equipment (Biorad®).

### Statistical analysis

Genotypic and allelic frequencies were obtained by direct counting. The mean was computed as the arithmetic average, obtained by summing all observed values and dividing by the total number of observations. Data dispersion was characterized using the standard deviation (SD), which quantifies the variability of individual observations around the mean.

## Results

A total of 103 blood donors, comprising 37 (36%) females and 66 (64%) males, with a mean age of 34 ± 9 years, were included in this study. The samples were collected between October and December 2016 from the Blood Bank of Brasília, Central-West Brazil. Ethnic self-declaration classified most donors as European descendants (67%), followed by mixed race (30%) and African descendants (7%).

Routine serological D typing revealed weak or discrepant agglutination results in 1.25% of the D+ samples. Analysis of the RhCE profile (C/c and E/e antigens) revealed a predominance of the Dccee phenotype (67.0%), followed by Ccee (21.4%), ccEe (6.8%), CCee (3.9%), and CcEe (1.0%) (Table 1). Serological testing with different anti-D reagent clones revealed agglutination scores of <2+ in most samples. Additionally, 27% of the samples were classified as inconclusive, and 21% exhibited RhD serological discrepancies (Table 2).

**Table 1:** RhCE phenotypic frequencies.

Donors (n)	RhCE phenotypes	Probable genotypes	Frequency
69	D ccee	*Dce/dce*	67.0%
22	D Ccee	*DCe/dce*	21.4%
7	D ccEe	*DcE/dce*	6.8%
4	D CCee	*DCe/dCe*	3.9%
1	D CcEe	*DCe/dcE*	1.0%

**Table 2:** Agglutination reaction profiles in direct and indirect D typing.

	Monoclonal Antibodies				
Reactivity	TH-28(IgM)	MS-201/MS-26 (IgM/IgG)	ESD1 IgG (AGH)	N°	%
Discrepancy	0	0	1+/3+/4+	22	21.4
<2+	1+	1+	2+/3+/4+	53	51.5
Inconclusive	0/+^w^	0/+^w^	3+/4+	28	27.2

*RHD* allele-specific PCR and sequencing identified weak D polymorphisms in 94 (91%) samples and *RHD* genotypes encoding partial D antigens in four samples (3.9%) (Table 3). No alterations were detected in the gene regions analyzed of two (1.94%) samples. Furthermore, three samples (2.91%) showed no amplification in at least two of the ten *RHD* exons, suggesting the presence of partial *RHD* alleles and/or hybrid genes.

**Table 3:** Distribution of RHD variant alleles.

*RHD* allele	Nucleotide change	Exon	Amino acid change	n	Frequency (%)
RHD*01W.1 RHD*weak D type 1	c.809 T>G	6	Val270Gly	2	1.94%
					
RHD*01W.2 RHD*weak D type 2	c.1154 G>C	9	Gly385Ala	6	5.83%
					
RHD*01W.3 RHD*weak D type 3	c.8 C>G	1	Ser3Cys	17	16.5%
					
RHD*DAR1.2 RHD*weak D type 4 *(weak D 4.2.2)*	c.602 C>G c.667 T>G c.744 C>T c.957 G>A c.1025 T>C	4, 5, 7	Thr201Arg	33	32.0%
					
RHD*DAR3.1 RHD*weak D type 4 (weak partial D 4.0)	c.602 C>G c.667 T>G c.819 G>A	4, 5, 6	Thr201Arg	34	33.0%
					
RHD*01W.38 RHD*weak D type 38	c.833 G>A	6	Gly278Asp	1	0.97%
RHD*01W.145 RHD*weak D type 145	c.41 C>G	1	Pro14Arg	1	0.97%
RHD*07.01 RHD*DVII.1	c.329 T>C	2	Leu110Pro	2	1.94%
RHD*12.02 RHD*DOL 1	c.509 T>C c.667 T>G	4,5	Met170Th Phe223Val	1	0.97%
RHD*12.02 RHD*DOL 2	c.509 T>C c.667 T>G c.1132C>G	4,5,8	Met170Thr Phe223Val Leu378Val	1	0.97%

The integration of molecular and serological data enabled association analyses with RhCE phenotypes (Table 4). The most common phenotype associated with the identified variants was Dccee. Notably, the RHD*weak D type 3 allele, typically associated with the Ccee phenotype, was also found in samples with ccee and CCee phenotypes. Similarly, the RHD*weak partial 4 allele, which is normally associated with the ccee phenotype, was also observed in individuals with Ccee and CcEe phenotypes. All other associations were consistent with those described in the literature.

**Table 4:** RHD variant alleles and RhCE phenotypes.

*RHD* allele	RhCE Phenotype	Donors (n)
RHD*weak D type 1	Ccee	2
RHD*weak D type 2	ccEe	6
RHD*weak D type 3	ccee CCee Ccee	1 1 15
RHD*weak D type 4 *(weak partial D 4.0)*	ccee Ccee CcEe	32 1 1
RHD*weak D type 4 *(weak partial D 4.2.2)*	ccee Ccee ccEe	31 1 1
RHD*weak D type 38	Ccee	1
RHD*weak D type 145	ccee	1
RHD*DVII	Ccee	2
RHD*DOL1	ccee	1
RHD*DOL2	ccee	1
Conventional *RHD*	CCee	2
*RHD* allele not classified	ccee CCee	2 1

Further analysis revealed that all samples with RHD*weak D type 1 and RHD*weak D type 2 alleles showed no reactivity with monoclonal antibodies in tube agglutination tests. Some alleles, including RHD*weak partial 4, RHD*weak D type 38, and RHD*weak D type 145, produced negative results in direct microplate phenotyping but were reactive in the IAT. Both the RHD*weak D type 38 and the RHD*weak D type 145 alleles showed weak reactivity (1+) in the IAT. Additionally, the RHD*weak D type 3 and RHD*weak partial 4 alleles consistently met all three defining criteria for this study: serological discrepancy, weak agglutination (<2+), and inconclusive results (Table 5).

**Table 5:** Correlation between *RHD* genotypes and serological anti-D reactivity.

		Monoclonal antibodies x agglutination reactivity			
		Microplate (Direct typing) RT			Gel (IAT)
*RHD* allele	n	TH-28(IgM)	MS-201/MS-26 (IgM/IgG)		ESD1(IgG)
RHD*weak D type 1	2	0	0		3+
RHD*weak D type 2	6	0	0		3+
RHD*weak D type 3	9 5 3	1+ 0/+^w^ 0	1+ 0/+^w^ 0		2+/3+ 3+ 3+
RHD*weak D type 4 (weak partial D 4.0)	31 2 1	1+ 0/+^w/1+^ 0	1+ 0/+^w/1+^ 0		3+/4+ 3+/4+ 3+
RHD*weak D type 4 *(weak partial D 4.2.2)*	12 12 9	1+ 1+ 0	1+ 0/+^w/1+^ 0		3+/4+ 3+/4+ 2+3+
RHD*weak D type 38	1	0	0		1+
RHD*weak D type 145	1	0	0		1+
RHD*DVII	2	1+	1+		3+/4+
RHD*DOL 1	1	0	2+		3+
RHD*DOL 2	2	0	1+		3+
Conventional *RHD*	2	1+	1+		3+
*RHD allele* not classified	1 1 1	0 1+ 1+	0 1+ 0/+^w/1+^		3+ 3+ 3+

⁎ IAT: Indirect antiglobulin test; n: Number of individuals; w: Weak agglutination reaction; rt: Room temperature

## Discussion

This study conducted a molecular characterization of variant D antigens in 103 samples collected from blood donors in the Central-West region of Brazil. The frequency of atypical serological D reactions was 1.25%, differing from findings in other regions of Brazil. According to the literature, the frequency of D-positive samples presenting weak or discrepant agglutination during serological D typing ranges from 0.3% to 0.8% in cohorts from Southeastern Brazil. For example, studies conducted in São Paulo reported frequencies of 0.3% [15], 0.52% [22], 0.8% [23], and 0.79% [14]. In contrast, a study from Paraná reported a significantly higher frequency of variant D antigens at 9.75% [16].

These discrepancies may be attributed to differences in the quality of anti-D reagents (clones) and the methodologies used [24,25,8,26] For instance, Campos et al. used hemagglutination using gel cards and conventional tube methods with anti-D IgM (clone P3 × 61) and anti-D blends (clones P3 × 290, P3 × 35, P3 × 61, P3 × 2123B10) (Grifols, Spain) for gel testing, and anti-D IgM (clone MS201) and anti-D IgG (clone MS26) from Fresenius Kabi, Brazil for tube testing [15]. Arnoni et al. [22] employed the solid-phase method using IgM clones RUM-1 and the D175 (IgM) + D415 (IgG) blend (Immucor Inc.). Cruz et al. [23] and Rodrigues et al. [21] used tube methodologies with monoclonal anti-D blends (clones MS26/MS201) and anti-D IgG (MS26). Zacarias et al. [16], like the present study, utilized microplate hemagglutination with monoclonal anti-D IgM anti-DVI- (TH28) and anti-DVI+ (MS201/MS26, IgM/IgG) reagents (Biorad., Brazil). Additionally, regional differences in the prevalence of atypical serological D reactions may be influenced by Brazil’s diverse ethnic composition and historical colonization patterns. The distribution of *RHD* variants is known to vary significantly with race and ethnicity [8].

The serological testing in this study revealed that 21.4% of samples did not react with either of the two anti-D reagents (clones TH-28 and MS26/MS201) used in direct hemagglutination. Furthermore, 22.7% reacted with only one of the two reagents and required further confirmation of the D antigen phenotype using the IAT at 37°C. Without this confirmation step, these samples might have been misclassified as D-negative. This finding underscores the importance of confirming weak D phenotypes during routine immunohematological screening, particularly for blood donors to minimize the risk of anti-D alloimmunization in D-negative recipients.

Molecular characterization of the *RHD* gene revealed that most samples (67/103; 65%) were classified within the RHD*weak partial 4 cluster, followed by the RHD*weak D type 3 cluster (17/103; 16%). Lower frequencies were observed for RHD*weak D type 1 (2/103; 1.9%) and RHD*weak D type 2 alleles (6/103; 5.8%). These findings are consistent with Cruz et al. [23], who also identified RHD*weak partial 4 as the most prevalent variant in Brazilian blood donors. Arnoni et al [22] similarly reported a 45% frequency (191/421) of the RHD*weak partial 4 cluster in Southeastern Brazilian donors. Dezan et al. [29] further reported a higher prevalence of RHD*DAR and RHD*weak D-type 38 alleles in São Paulo. In contrast, Campos et al. [15] identified higher frequencies of weak D types 1 (70/223), 2 (44/223), and 3 alleles (26/223). Another study, evaluating 306 samples from patients and donors across Brazil, reported frequencies for the RHD*weak D type 1 (76/306) and type 2 alleles (75/306) [27].

RHD*weak types 1, 2, and 3 alleles, known as the “European cluster,” predominate in 90% of D variant cases in Caucasian populations. In contrast, the RHD*weak partial 4 cluster, which occurs in <2% of European individuals, is more common in people of African ancestry [19,15]. The high frequency of RHD*weak partial 4 alleles found in the present cohort reflects the strong African genetic influence in the Central-West, particularly Brasília. This is consistent with previous studies showing that RHD*weak partial 4 clusters are largely restricted to individuals of African descent [28,29,30,31].

This study also identified the RHD*weak D type 38 and weak D type 145 *RHD* variant alleles. The RHD*weak D type 38 allele is characterized by low antigenic density (60–151 sites per cell) and may be undetectable by standard serological methods unless specialized techniques like adsorption and elution are used [32,33]. This variant was not detected by monoclonal anti-D during direct typing or the IAT phase in the present study. Previously, the RHD*weak D type 38 allele was associated with the DCe haplotype, and occurs at a low frequency (1.5%) in Caucasians [34], although other studies reported frequencies ranging from 1.2% to 19.81% [15,22,32]. The RHD*weak D type 145 allele was first described in a Brazilian blood donor; this allele was found in association with the Dce haplotype. No reactivity was observed in direct hemagglutination with monoclonal anti-D antibodies, but agglutination scores reached 1+ in the IAT phase. All donors identified with RHD*weak D type 38 and RHD*weak D type 145 were appropriately informed and managed with caution due to their potential to induce anti-D alloimmunization. A conservative clinical approach was adopted, whereby these individuals were considered RhD-negative for transfusion and Rh prophylaxis purposes. Conversely, when acting as blood donors, they were classified as RhD-positive. This dual management strategy represents a pragmatic and safety-oriented approach that minimizes the risk of alloimmunization while preserving the availability of RhD-positive blood components.

A classification into the RHD*weak partial 4 cluster revealed the presence of RHD*DAR3.1 (33%) and RHD*DAR1.2 (32%). The RHD*DAR allele is particularly significant due to the loss of at least nine of the 37 RhD epitopes, resulting in markedly reduced antigen expression [24]. RHD*DAR is part of the RHD*weak partial 4 cluster and is typically associated with the Dce haplotype [28,30], a finding corroborated in the present study.

The DOL1 and DOL2 variants, also linked to the Dce haplotype, are common in individuals of African descent [35,36]. These variants show an antigen density of approximately 4,700 sites per cell [37] and have been implicated in anti-D alloimmunization. The DVII variant lacks one RhD epitope (epD8) and expresses low antigen density (3,600 to 8,400 sites per cell, placing individuals at risk for anti-D development after transfusion or pregnancy [38]. The frequencies observed in this study are in agreement with previous data, which estimate frequencies at around 2% [3,27].

As confirmed here and in earlier reports, conventional serological methods cannot reliably differentiate between weak D and partial D phenotypes, as both present similar reactivity. In cases of inconclusive D typing, the choice of high-quality reagents is critical, as discrepancies between IgM and IgM/IgG blends are well documented.

For more precise identification of *RHD* variants, however, additional analysis of other regions of the *RHD* gene is necessary, as many variants share the same polymorphisms. Advanced molecular tools, such as large-scale genotyping platforms (BeadChip, BloodChip, microarrays), offer high-throughput alternatives capable of simultaneously detecting multiple RBC antigens [39,40]. Nevertheless, the high costs associated with these methods limit their routine use in Brazilian blood banks. Therefore, the development of an *RHD* genotyping platform tailored to the genetic diversity of the Brazilian population, using cost-effective technologies, is crucial to improving transfusion safety and accuracy nationwide.

## Author contributions

ALAM performed the investigation and data curation and wrote the original draft. WRM, BMSP, and FGUA samples, resources, and methodology analysis. FLSS and LC supervision, review & editing. DTC, SK, and ESR conceptualization, formal analysis, funding acquisition, review, and editing.

## Funding information

This work was supported by Fundação de Amparo à Pesquisa do Estado de São Paulo (FAPESP) (Process number 2017/26950-6), the Conselho Nacional de Desenvolvimento Científico e Tecnológico, Brazil (422118/2016-8), (INCTC-465539/2014-9), and Fundação Hemocentro de Ribeirão Preto (FUNDHERP).

## Data availability statement

The data that support the findings of this study are available from the corresponding author upon reasonable request.

## Conflicts of interest

There are no conflicts of interest.

## Appendix Supplementary materials

Image, application 1
