# Supplementary material for: Genetic diversity of the RHD gene in blood donors from the Brazilian Central-West
Source: Hematol Transfus Cell Ther. 2026 Jul 28;48(4):106506. doi: 10.1016/j.htct.2026.106506 (PMC13448903; doi:10.1016/j.htct.2026.106506)
Supplement: Supplementary file 1 [file mmc1.docx]

**Supplementary Figure 1:** Serological phenotyping and *RHD* genotyping. (A) Direct phenotyping of ABO and RhD antigens using a microplate assay. (B) Polymerase chain reaction (PCR) amplification of *RHD* intron 4 (498 bp) and exon 7 (95 bp) regions. (C) Detection of the *RHD* pseudogene (*RHDΨ*) by PCR amplification of a 250-bp fragment. (D) Detection of RHD weak D type 1 (c.809T>G) by PCR amplification of a 478-bp fragment followed by restriction enzyme digestion with Alw44I (ApaLI), characterizing the target RHD* weak D type 1 allele by the generation of 175-bp and 303-bp fragments. (E) Detection of RHD* weak D type 2 (c.1154G>C) by allele-specific PCR amplification of a 126-bp fragment. (F) Detection of RHD* weak D type 3 (c.8C>G) by allele-specific PCR amplification of a 166-bp fragment. (G) Detection of RHD* weak/partial D type 4 (c.602C>G) by allele-specific PCR amplification of a 138-bp fragment. The *human growth hormone* (*HGH*) gene (434 bp) was co-amplified in all reactions as an internal control for DNA quality. Positive controls and no-template controls (NTC) were included in all assays. PCR products were resolved on a 2% agarose gel stained with GelRed. A molecular weight marker (Mid Gel Pilot Mid-Range Ladder, 100 bp; Qiagen) was used for fragment size estimation.

**Supplementary Figure 2:** *RHD* gene sequencing. Partial electropherograms from Sanger sequencing showing the polymorphisms identified after amplification of exons 1–10 of the *RHD* gene and subsequent sequencing reaction.
